# Effects of methylphenidate on children with attention deficit hyperactivity disorder: a study using clinical and multimodal approaches including go/no-go task and functional near-infrared spectroscopy

**DOI:** 10.3389/fphar.2025.1726716

**Published:** 2025-12-08

**Authors:** Fang Shen, Hui Zhou

**Affiliations:** Department of Rehabilitation Medicine, Key Laboratory of Birth Defects and Related Diseases of Women and Children, Ministry of Education (MOE), West China Second University Hospital, Sichuan University, Chengdu, Sichuan, China

**Keywords:** attention deficit hyperactivity disorder, methylphenidate, go/no-go task, response inhibition, functional near-infrared spectroscopy

## Abstract

**Objective:**

This study aims to provide an objective evaluation of the therapeutic effects of methylphenidate (MPH) in children with attention deficit hyperactivity disorder (ADHD) by integrating clinical assessments, the go/no-go task, and functional near-infrared spectroscopy (fNIRS).

**Methods:**

This study employed a two-part design. First, a cross-sectional comparison was conducted between 36 children with ADHD and 12 typically developing controls (TDC) to establish baseline neurocognitive and functional differences. Second, a pre-post intervention design was used to evaluate the effects of a 6-month MPH treatment on the 36 children with ADHD. All participants underwent clinical assessments, including the Swanson, Nolan, and Pelham IV Rating Scale (SNAP-IV) and the Weiss Functional Impairment Rating Scale (WFIRS), a go/no-go task, and fNIRS to measure average oxygenated hemoglobin (Δavg oxy-Hb) changes in targeted brain regions, including the medial prefrontal cortex (mPFC), dorsolateral prefrontal cortex (DLPFC), and temporal lobe (TL).

**Results:**

The findings of the present study revealed that, at baseline, children with ADHD had significantly higher scores on the SNAP-IV and WFIRS than the TDC group. On the go/no-go task, accuracy for both go and no-go trials was significantly lower in the ADHD group than in the TDC group. Furthermore, fNIRS data indicated that the ADHD group had significantly lower Δavg oxy-Hb levels than the TDC group in the bilateral mPFC, right DLPFC, and bilateral TL. After 6 months of MPH treatment, children with ADHD showed significant reductions in SNAP-IV and WFIRS scores. Concurrently, they showed significantly increased accuracy on both go and no-go trials, along with significantly decreased reaction times on go trials. Furthermore, Δavg oxy-Hb levels increased significantly in the right mPFC.

**Conclusion:**

Our findings suggest that activation in the right mPFC is a viable objective neurofunctional biomarker for monitoring MPH treatment response using fNIRS. Concurrently, a multimodal assessment strategy integrating clinical evaluations, neuropsychological testing (go/no-go task), and fNIRS holds significant promise as a robust method for objectively determining the therapeutic efficacy of MPH in pediatric ADHD.

## 1 Introduction

Attention deficit hyperactivity disorder (ADHD) is a chronic neurodevelopmental disorder with onset in childhood that can persist into adolescence and adulthood. Its core symptoms are age-inappropriate inattention, hyperactivity, and impulsivity (Rajaprakash and Leppert, 2022), which severely impair the patient’s academic performance, occupational development, and social interactions. Epidemiological studies indicate that the global prevalence of ADHD in children has reached 8.0% (Ayano et al., 2023), with a significant gender difference: the incidence in males is approximately 2–3 times higher than in females. The latest survey data from China show a prevalence of approximately 6.4% among children and adolescents (Li et al., 2022), with symptoms persisting into adolescence for about 70% of patients and extending into adulthood for 30%–50%. Simultaneously, co-occurrent conditions are also common, including affective disorders, substance use disorders, and other neurodevelopmental disorders (Gnanavel et al., 2019). ADHD is associated with a wide range of adverse outcomes. At the individual level, it is linked to educational and occupational failure, teenage pregnancies, legal offenses, road accidents, and increased mortality. At the societal level, it contributes to substantial economic costs stemming from income loss, healthcare expenditures, and the need for educational support (Faraone et al., 2021). Therefore, ADHD is a significant burden for the affected youngsters, adults, their families and society everywhere.

Individuals with ADHD often exhibit neuropsychological deficits, particularly in executive functions such as attention, response inhibition, planning, and working memory (Faraone et al., 2021). Specifically, deficits in working memory are observed in approximately 75%–85% of adolescents with ADHD, while impairments in inhibitory control and cognitive flexibility are reported in 21%–46% and 10%–38% of this population, respectively (Kofler et al., 2019). However, Barkley posited that a deficit in inhibitory control constitutes the core executive dysfunction in ADHD (Barkley, 1997). This core deficit is thought to cascade and contribute to secondary dysfunctions in other executive domains, including working memory. Therefore, while working memory deficits are indeed highly prevalent, they are often conceptualized as a downstream consequence of primary inhibitory failures. Inhibitory control refers to a person’s ability to overcome automatic or strong responses to stimuli. Inhibition ability deficiency can lead to a decrease in an individual’s ability to respond to external stimuli, which may impair functional independence and safety, and is known as a hallmark of executive dysfunction in ADHD patients (Chmielewski et al., 2019). Inhibitory control encompasses response inhibition and interference control (Chevalier et al., 2020). Response inhibition refers to the ability to inhibit responses to behaviors that are incongruent or conflicting with current goals (Cheng et al., 2025). Interference control describes the ability to selectively attend and resist distractions at the level of perception (Mueller et al., 2017). Response inhibition impairments are widely recognized as a core deficit of ADHD (Bari and Robbins, 2013). Moreover, signaling in the prefrontal cortex (particularly the inferior frontal cortex and the ventrolateral prefrontal cortex) and the basal ganglia is associated with these impairments (Aron et al., 2014; Aron et al., 2014). The go/no-go paradigm is a primary neuropsychological tool for the objective assessment of response inhibition (Baijot et al., 2017; Breitling-Ziegler et al., 2020; Kolodny et al., 2022; Ogrim and Kropotov, 2019). It requires participants to execute a rapid motor response to a designated stimulus (e.g., a “go” cue) while inhibiting that response to a different stimulus (e.g., a “no-go” cue), thus providing a measure of response inhibition. Specific performance metrics serve as indices for core ADHD symptoms. For instance, the error rate on “go” trials is widely interpreted as a measure of inattention, while the error rate on “no-go” trials and the reaction time on “go” trials are commonly viewed as indicators of impulsivity (i.e., impaired response inhibition) (Münger et al., 2021).

Methylphenidate (MPH) stands as the first-line pharmacological intervention for ADHD in children aged six and above. A primary mechanism underlying its therapeutic effect involves the augmentation of synaptic dopamine (DA) and norepinephrine (NE) levels within the prefrontal cortex and striatum, a process that is instrumental in ameliorating the core symptoms of ADHD and enhancing specific cognitive capabilities (Cortese et al., 2018; Faraone, 2018). Additionally, pharmacological treatment effectively improves outcomes and quality of life (Bellato et al., 2025). Although the synaptic mechanisms of action have been extensively studied *in vitro* and in animal models (Zimmer, 2017), it remains unclear how these neurochemical changes translate into beneficial clinical and behavioral effects. At present, the assessment of MPH’s efficacy in the pediatric and adolescent ADHD population is predominantly contingent upon indirect measures, including subjective reports from parent and teacher rating scales, as well as performance on neuropsychological tests (Catalá-López et al., 2017; Cortese et al., 2018; Cortese et al., 2017a; Maneeton et al., 2015; Padilha et al., 2018; Storebø et al., 2015; Storebø et al., 2023). While neuropsychological tests can rapidly generate performance-based indices of response inhibitory function, they lack the capability to localize the underlying neural substrates. Moreover, a single test of response inhibitory function fails to delineate the specific brain networks that are recruited by individuals with ADHD during the engagement of this cognitive process. This research gap makes it difficult for clinicians and patients to reach a clear consensus on the therapeutic benefits of MPH. Therefore, there is an urgent need for biomarkers that can objectively reflect the therapeutic effects of MPH.

Neuroimaging techniques, such as functional Magnetic Resonance Imaging (fMRI) and functional Near-Infrared Spectroscopy (fNIRS), are powerful tools for bridging this gap by investigating the effects of pharmacological treatment on the brain at a systems-level. While fMRI is considered the gold standard, its clinical utility in the pediatric ADHD population is constrained by significant drawbacks, including prohibitive costs, extreme susceptibility to motion artifacts, prolonged scan durations, and challenges with child compliance. In contrast, fNIRS presents a compelling alternative, characterized by its high temporal resolution, enhanced practicality, and relative insensitivity to motion. This makes fNIRS particularly well-suited for studying children with ADHD, who often exhibit suboptimal compliance. By enabling the design of tailored task paradigms that align with research goals, fNIRS can be integrated with neuropsychological assessments to effectively elucidate the task-specific brain network dynamics in this population (Cui et al., 2024; Inoue et al., 2012; Kaga et al., 2020; Kurane et al., 2024; Li et al., 2021; Li et al., 2025; Miao et al., 2017; Monden et al., 2015; Sutoko et al., 2019; Wu et al., 2023; Zhu et al., 2023).

Currently, the assessment of methylphenidate’s effects in children with ADHD primarily relies on subjective rating scales to evaluate behavioral changes in daily life and neuropsychological tasks to measure alterations in cognitive performance. However, neither method can directly reveal the neurofunctional alterations in the brain induced by methylphenidate. These neurofunctional changes constitute the biological foundation for the observed behavioral and cognitive improvements. This critical gap serves as the fundamental rationale for introducing a multimodal objective assessment in our study. We need an integrated evaluation framework that spans different levels: “neural mechanisms, cognitive behavior, and clinical symptoms.” fNIRS can directly and non-invasively measure neural activity levels in key brain regions associated with executive function during task performance. This provides objective neurophysiological evidence for the improvement in cognitive behavior. The integration of these methods links subjective clinical impressions, objective cognitive-behavioral performance, and underlying neural mechanisms, thereby establishing a more comprehensive and convincing objective evaluation system for MPH’s therapeutic efficacy.

Consequently, this study integrates clinical assessments, neuropsychological testing, and fNIRS to investigate the therapeutic effects of MPH on children with ADHD, identify neurobiological biomarkers associated with treatment response, and thereby provide an empirical foundation for objective clinical evaluation.

## 2 Materials and methods

### 2.1 Participants

Thirty-six children diagnosed with ADHD (aged 6–14 years) and twelve typically developing children (TDC) were enrolled from the outpatient service of the Department of Pediatric Rehabilitation at the Second Affiliated Hospital of Sichuan University. To ensure comparability between the groups and to mitigate potential confounding variables, participants in the ADHD and TDC cohorts were individually matched based on age (6–14 years), sex, and full-scale intelligence quotient (FSIQ). Exclusively right-handed individuals were included in the study. FSIQ was evaluated with the Chinese version of the Wechsler Intelligence Scale for Children-Fourth Edition (WISC-IV), with enrollment restricted to those exhibiting an FSIQ of 80 or higher.

The diagnosis for all children in the ADHD cohort was established by a qualified pediatric psychiatrist based on the diagnostic criteria outlined in the Diagnostic and Statistical Manual of Mental Disorders, Fifth Edition (DSM-5). To evaluate clinical symptoms and associated functional impairments in daily life, the parent versions of the Swanson, Nolan, and Pelham IV Rating Scale (SNAP-IV) and the Weiss Functional Impairment Rating Scale (WFIRS) were administered. Concurrently, behavioral performance metrics and average oxygenated hemoglobin (Δavg oxy-Hb) concentrations were acquired via fNIRS as the ADHD children performed the go/no-go task. The TDC were recruited through a school-based screening process, and a thorough history confirmed the absence of any neurological, psychiatric, or developmental disorders.

Participants were excluded based on the following criteria: (1) FSIQ below 80 as measured by the WISC-IV, or failure to complete the testing protocol owing to significant difficulties with comprehension or behavioral compliance; (2) any prior or current history of pharmacological treatment (e.g., stimulants) or non-pharmacological interventions for ADHD; (3) the presence of comorbid severe psychiatric conditions, such as schizophrenia; (4) documented organic brain disease, significant medical illness, or any genetic/metabolic disorder (e.g., epilepsy, congenital malformations, Down syndrome); (5) a concurrent diagnosis of other neurodevelopmental disorders, including autism spectrum disorder (ASD) or intellectual disability (ID); (6) a history of substance abuse, encompassing the use of psychoactive medications for non-medical purposes or illicit drugs; (7) withdrawal from the study or being lost to follow-up.

The study protocol was conducted in accordance with the Declaration of Helsinki and received approval from the Institutional Review Board (IRB) of West China Second University Hospital, Sichuan University. Prior to enrollment, written informed consent was secured from the parents or legal guardians of all participating children.

### 2.2 Intervention measures

Participants in the ADHD cohort received an oral regimen of MPH (Manufacturer: Xian Janssen Pharmaceutical Ltd.; NMPA Approval No.: HJ20170263; Strength: 18 mg tablets, 15 per pack). The pharmacotherapy was initiated at 18 mg/day and subsequently titrated up to a target dose of 36 mg/day (with a maximum allowable dose of 54 mg/day), guided by functional evaluation. The medication was prescribed for once-daily morning administration over a 6-month consistent treatment duration. Post-treatment, the participants’ clinical symptomatology and functional deficits in daily life were re-evaluated utilizing the parent-rated SNAP-IV and the WFIRS. Concurrently, fNIRS was employed to acquire behavioral metrics and Δavg oxy-Hb concentrations from the ADHD group while they performed the go/no-go task.

**FIGURE 1:**
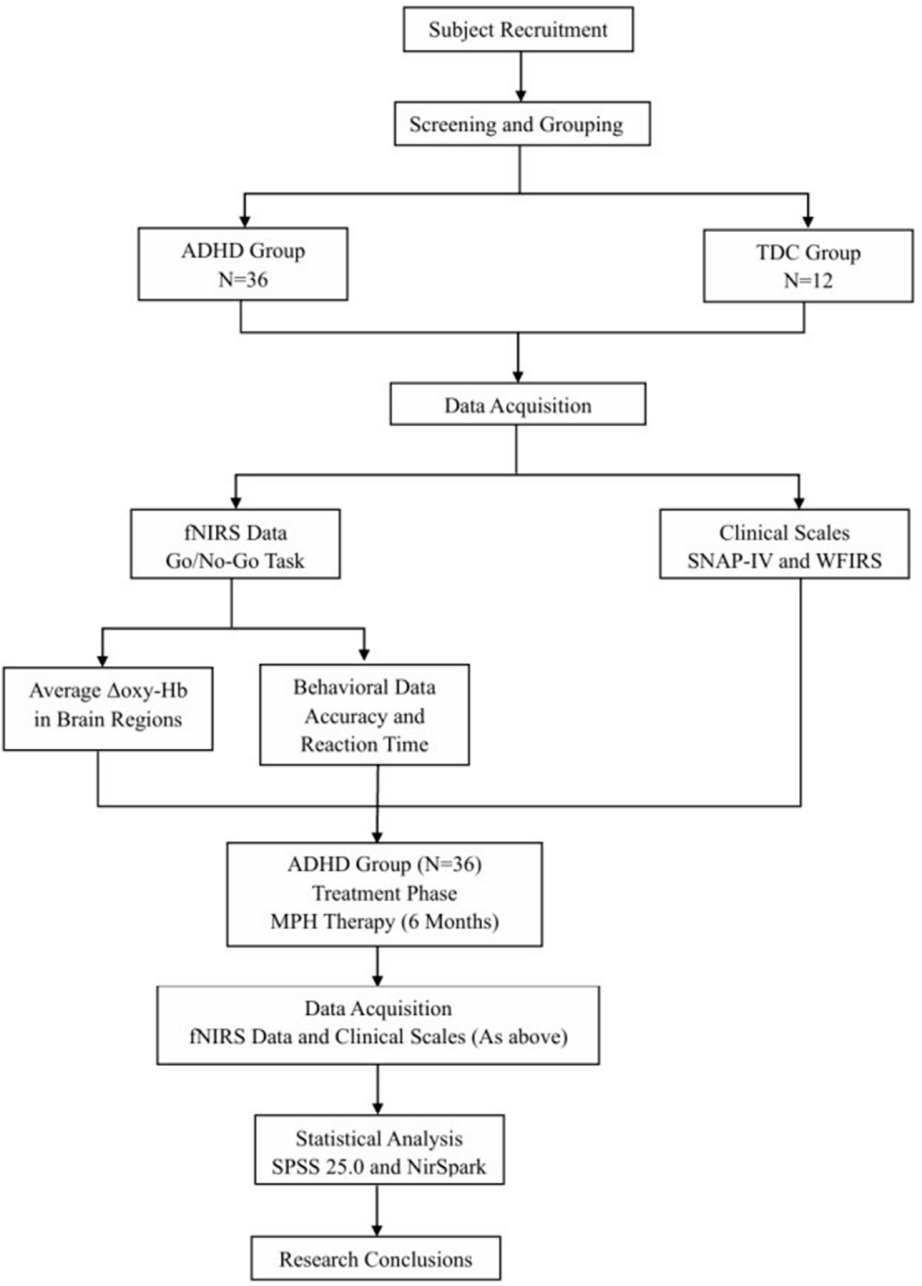
Experimental design flowchart. ADHD, attention deficit hyperactivity disorder; TDC, typically developing children; MPH, methylphenidate; fNIRS, functional Near-Infrared Spectroscopy; SNAP-IV, Swanson, Nolan, and Pelham IV Rating Scale; WFIRS, Weiss Functional Impairment Rating Scale.

No pharmacological interventions were administered to the TDC group. Under the supervision of a clinician, parents completed the parent-rated SNAP-IV and the WFIRS to assess their children’s core symptomatology and functional impairment. In parallel, fNIRS was employed to acquire behavioral performance metrics and Δavg oxy-Hb concentration in the TDC during the go/no-go task. Based on practical considerations and ethical principles, data from the healthy control group were collected only once. This approach was taken to reduce the burden on healthy participants and to address the challenges associated with multiple testing sessions. Figure 1 illustrates the experimental protocol used in this study.

### 2.3 Experimental design

**FIGURE 2:**
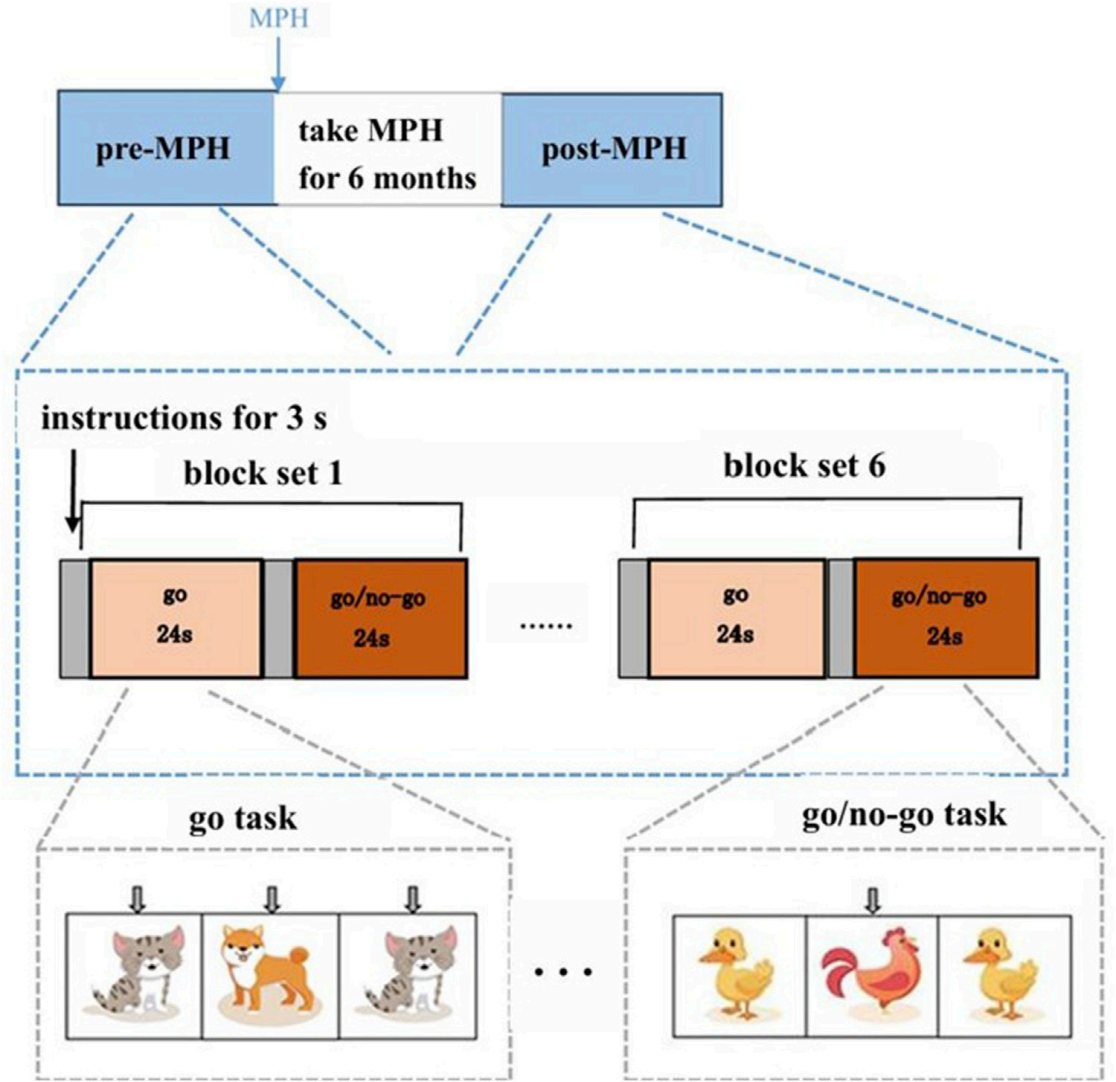
Experimental design. Brain activity was measured with fNIRS measurements during the go/no-go task.

Go/no-go task was generated by E-Prime2.0 and presented in a 17″ tablet computer screen. The distance between the subject’s eyes and the screen was ∼50 cm (J. Wang et al., 2024). The detailed experimental protocol is depicted in Figure 2. The ADHD cohort performed the task on two occasions: a baseline session before MPH initiation and a follow-up session 6 months post-administration. In contrast, the TDC cohort performed the task on a single occasion. The paradigm comprised 6 blocks, each pairing a go task (serving as the baseline condition) with a go/no-go task (the target condition). Each task block was preceded by a 3-second cue. The duration of each task was 24 s, during which 24 stimuli were presented, culminating in a total task length of 324 s. For the go task, participants were instructed to respond by pressing a button to cartoon images of a “cat” or a “dog.” Conversely, for the go/no-go task, the rule was to respond to a “chicken” (go trial) but to inhibit the response to a “duck” (no-go trial). Overall, 50% of all trials were no-go trials, and the sequence of stimulus presentation was pseudo-randomized. Equal numbers of “go” and “go/no-go” pictures were presented to decrease the likelihood of changes in cerebral activity between the two groups as shown earlier (Liddle et al., 2001). To ensure task comprehension, a practice session was conducted under the examiner’s supervision before the formal experiment commenced. Participants were also instructed to maintain quietude during data acquisition. The reaction time (RT) and accuracy rate were recorded for each trial (J. Wang et al., 2024; Wu et al., 2023).

### 2.4 fNIRS data acquisition

**FIGURE 3:**
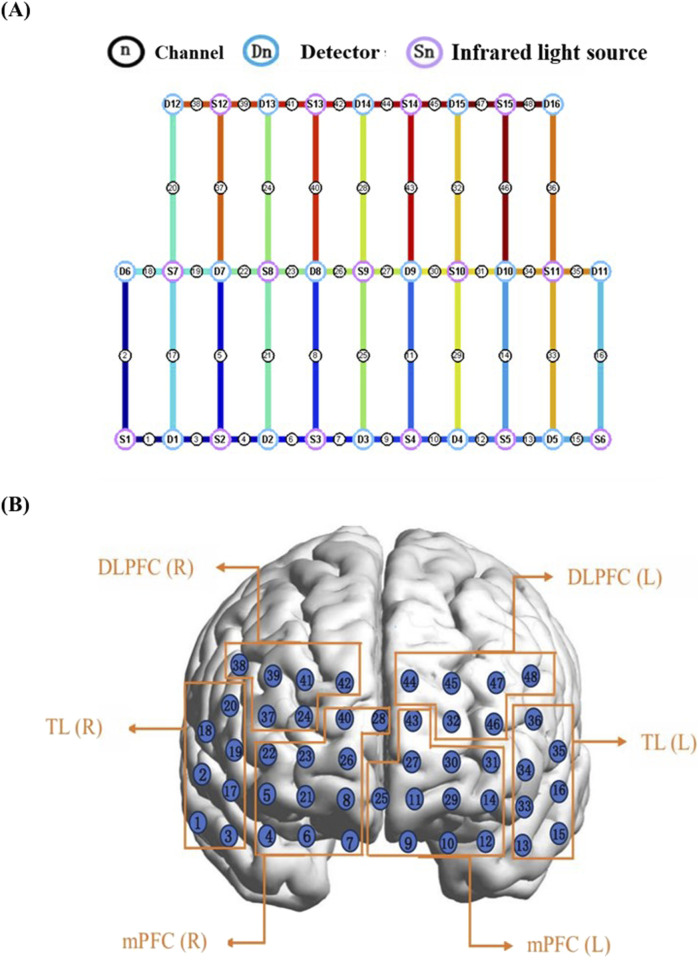
fNIRS measurement images. **(A)** Shows the positions of light sources and detectors as well as fNIRS channels, respectively, where S1–S15 are 15 infrared light sources, D1–D16 are 16 detectors and 1–48 indicate 48 fNIRS channels (CH 1–48); **(B)** ROI division diagram. DLPFC, dorsolateral prefrontal cortex; mPFC, medial prefrontal cortex; TL, temporal lobe; R, right; L, left.

The fNIRS data in this study were acquired using a NirScan system (Huichuang Medical Equipment Co., Ltd., Danyang, China). The system operates at three distinct wavelengths (730 nm, 808 nm, and 850 nm) with a sampling rate of 11 Hz. The participants wore a probe cap including 15 infrared light sources and 16 detectors placed on the scalp according to the international 10–20 system (D3 corresponds to FPZ) (Figure 3A). These probes formed 48 fNIRS channels which covered the frontal and temporal cortical areas (Wang et al., 2023). The inter-optode distance was fixed at 3 cm, with each channel defined by a source-detector pair. The detailed probe configuration is depicted in Figure 3A. Placement was initiated by positioning the central probe of the lowest row at the FPZ location (D3 at FPZ), with the remaining probes arrayed to cover the left and right prefrontal cortices along the Fp1 and Fp2 coordinates. The anatomical localization of the channels was performed using the NirSpace software system (HuiChuang). This system allows for the point-by-point acquisition of spatial coordinates for each channel, which are then automatically converted to the standard Montreal Neurological Institute (MNI) space. Subsequently, the software automatically assigns each channel to its corresponding Brodmann area and generates a report detailing the brain region covered by each channel. The Brodmann area distribution of all fNIRS channels is provided in Supplementary Material.

Our rationale for regions of interest (ROI) selection was grounded in a substantial body of literature on ADHD’s pathological models and the pharmacodynamics of methylphenidate. A key ROI was therefore identified as the prefrontal cortex (PFC), particularly its dorsolateral (DLPFC) and medial (mPFC) subregions. The justification for this is that the PFC is the neural hub for executive functions—including inhibitory control and working memory—which are characteristically impaired in ADHD. This is further supported by neuroimaging evidence from fNIRS and fMRI, which has repeatedly documented hypoactivation in the PFC of children with ADHD during tasks requiring response inhibition (Hart et al., 2013; Hou et al., 2023; Kaga et al., 2020; Kim et al., 2023; Li et al., 2024; Miao et al., 2017; Monden et al., 2015). In addition, MPH blocks DA and NE reuptake by inhibiting their respective transporters in the striatum and prefrontal cortex, leading to elevated extracellular levels of both neurotransmitters, particularly within the prefrontal cortex (Groom and Cortese, 2022). This medication improves neurocognitive functions, including working memory, attention, and inhibitory control (Coghill et al., 2014; Czerniak et al., 2013). Meanwhile, the brain regions that showed increased activation during the inhibitory control task following MPH treatment were primarily located in the frontal lobe (Czerniak et al., 2013; Duffy et al., 2021; J. Li et al., 2021; Monden et al., 2012a; Monden et al., 2012b; Poliakova et al., 2023).

Furthermore, we selected the temporal cortex as another key ROI. This is because ADHD is not merely a deficit of prefrontal executive functions but rather a disorder of whole-brain network dysregulation, particularly involving an imbalance between the Default Mode Network (DMN) and the Executive Control Network (ECN). The temporal cortex serves as a critical component and connectivity hub for both of these networks. Focusing exclusively on the prefrontal cortex could therefore overlook the drug’s effects on the entire cognitive system. Examining this region enables us to assess the broader network effects of methylphenidate treatment (Kim et al., 2023). The 48 fNIRS channels were divided into six brain regions of interests (ROIs): the left DLPFC (channels CH32, 44–48), the right DLPFC(channels CH24, 37, 38, 39, 41, 42), the left mPFC (channels CH9–12, 14, 25, 27, 29–31,43), the right mPFC (channels CH4–8, 21–23, 26, 28, 40), the left TL (channels CH13, 15, 16, 33–36), and the rightTL (channels CH1–3, 17–20) (Figure 3B). fNIRS signals were recorded concurrently from the TDC group and the ADHD group (pre- and post-MPH treatment) while they performed the go/no-go task. To derive hemodynamic changes, the raw light intensity data collected by the detectors were converted into changes in Δavg oxy-Hb concentration by applying the modified Beer-Lambert law, which accounts for light absorption and scattering within the cortical tissue (Wang et al., 2024; Wu et al., 2023).

### 2.5 fNIRS data analysis

The fNIRS data collected during the go/no-go task underwent preprocessing via the Preprocess module within the NirSpark software suite. The preprocessing pipeline commenced with the identification and removal of motion artifacts through spline interpolation, for which the signal standard deviation and peak amplitude thresholds were set at 6 and 0.5, respectively. Subsequently, a band-pass filter (0.01–0.1 Hz) was applied to attenuate physiological noise originating from respiration and cardiac activity, as well as other high-frequency artifacts. The final stage of preprocessing involved scaling the pathlength differential factor to a range of −6 to 6. Subsequently, the raw optical density signals for oxy-Hb and deoxygenated hemoglobin (deoxy-Hb), recorded during the task performance in children, were transformed into concentration changes based on the modified Beer-Lambert law (Wu et al., 2023).

Block-averaged analysis of the go/no-go task data was performed using the BlockAvg module within the NirSpark software suite. The analysis commenced by calculating the Δavg oxy-Hb concentration for each channel and each subject across two epochs: the ‘go’ condition (14–24 s post-onset, designated as the baseline) and the “go/no-go” condition (4–24 s post-onset, representing the task period) (Nagashima et al., 2014a). The task-related hemodynamic response for each channel, denoted as Δavg oxy-Hb, was then derived by subtracting the mean oxy-Hb of the baseline (“go”) condition from the mean oxy-Hb of the task (“go/no-go”) condition. To obtain region-specific values, channels were averaged within each predefined ROI, yielding the Δavg oxy-Hb for each region of interest. Oxy-Hb was adopted as the primary metric in this study owing to its documented higher sensitivity, more favorable signal-to-noise ratio and excellent test-retest reliability (Hoshi, 2003; Strangman et al., 2002).

### 2.6 Behavioral data analysis

We calculated the accuracy for go and no-go trials and the mean RT for go trials within each go/no-go block for every participant. Subsequently, accuracy and RT scores were averaged across all blocks. These mean values were then statistically analyzed as detailed in the next section. The accuracy for both go and no-go trials was computed by dividing the number of correct responses (i.e., a button press on go trials and successfully withholding the response on no-go trials) by the total number of go trials in the block. Since omission errors (i.e., failures to respond to go trials) and commission errors (i.e., incorrect responses to no-go trials) are already reflected in the go and no-go accuracy rates, respectively, these error types were not analyzed separately.

### 2.7 Statistical analysis

Statistical analyses were performed using SPSS version 25.0 (IBM Corp., Armonk, NY, USA). Continuous variables are expressed as mean ± standard deviation (M ± SD), with statistical significance defined as P < 0.05. A chi-square test was used to compare the gender distribution between the TDC and ADHD groups. If any expected frequency was less than 5, Fisher’s Exact test was used instead. The normality of the data distributions for the TDC and ADHD groups was evaluated using the Shapiro-Wilk test. To compare the TDC and pre-treatment ADHD groups (inter-group differences), either an independent samples t-test (for normally distributed data with homogeneity of variance) or the Mann-Whitney U (M-W) test was utilized, depending on the results of the normality test. These analyses were conducted on SNAP-IV and WFIRS scores, behavioral metrics from the go/no-go task, and the Δavg oxy-Hb values within each ROI. For intra-group comparisons, assessing the effects of MPH treatment in the ADHD group (pre- vs. post-treatment), a paired-samples t-test or the Wilcoxon signed-rank test was applied to the same set of outcome measures. To account for multiple comparisons, the False Discovery Rate (FDR) correction was implemented across all statistical tests.

## 3 Results

### 3.1 Demographic and clinical characteristics

The demographic and baseline characteristics of the participants are summarized as follows. The ADHD group (N = 36) had a mean age of 9.333 ± 1.673 years, whereas the TDC group (N = 12) had a mean age of 10.167 ± 2.691 years. The groups were well-matched in terms of gender distribution, with the ADHD group comprising 29 males and 7 females, and the TDC group comprising 9 males and 3 females. There were no statistically significant differences between the TDC and ADHD groups in terms of age, gender, and FSIQ. Comprehensive demographic and clinical characteristics of the participants in both the TDC and ADHD groups are summarized in Table 1.

**TABLE 1:** Demographics of participants.

Demographic characteristics	TDC group	ADHD group		
	Mean (SD)	Mean (SD)	*t/Fisher/*M-W test	*P*
Age (years)	10.167 (2.691)	9.333 (1.673)	*Z* = −1.206	0.228^a^
Sex				0.695^b^
Male	9	29		
Female	3	7		
FSIQ	105.250 (12.425)	98.028 (10.278)	*t* = 2.001	0.051^c^

ADHD, attention-deficit hyperactivity disorder patient; TDC, typically developing children; SD, standard deviation; FSIQ: full-scale intelligence quotient.

^a^ Mann-Whitney U test.

^b^ Fisher’s Exact test.

^c^ Independent samples *t*-test.

The scale-based assessments of clinical symptoms and functional impairments are summarized in Table 2. At baseline, the parent-rated SNAP-IV revealed that the ADHD group exhibited significantly greater symptom severity than the TDC group across the inattention, hyperactivity/impulsivity, and oppositional defiant subscales. Subsequent to MPH treatment, children with ADHD demonstrated a significant reduction in scores on all three SNAP-IV subscales. Similarly, baseline WFIRS scores indicated significantly higher levels of functional impairment in the ADHD group compared to the TDC group in the family, learning and school, life skills, self-management, social activities, and adventure activities domains. Post-MPH treatment, significant improvements were observed, as evidenced by a marked decrease in scores across all six WFIRS functional domains.

**TABLE 2:** Clinical characteristics.

Clinical characteristics	TDC group	ADHD group					
		Pre-MPH	Post-MPH	TDC vs. Pre-MPH		Pre-MPH vs. Post-MPH	
	Mean (SD)	Mean (SD)	Mean (SD)	*t/*M-W test	*P*	*t/Wilcoxon test*	*P*
SNAP-IV							
Inattention	4.417 (3.528)	14.833 (4.814)	12.306 (3.717)	*t* = −6.884	<0.001	*t* = 4.009	<0.001
Hyperactivity/impulsivity	3.500 (2.844)	8.083 (4.500)	6.389 (2.930)	Z = −3.145	0.002	*Z* = −7.305	<0.001
Oppositional defiance	3.167 (0.937)	4.528 (1.874)	3.750 (1.888)	*t* = −3.293	0.002	*Z* = −6.651	<0.001
WFIRS							
Family	0.266 (0.303)	0.701 (0.349)	0.560 (0.303)	*Z* = −3.378	<0.001	*t* = 3.066	0.004
Learning and school	0.125 (0.142)	0.866 (0.347)	0.692 (0.311)	*Z* = −4.956	<0.001	*Z* = −6.211	<0.001
Life skills	0.483 (0.335)	0.924 (0.192)	0.814 (0.265)	*t* = −5.654	<0.001	*t* = 3.328	0.002
Self-management	0.194 (0.300)	0.655 (0.303)	0.553 (0.258)	*Z* = −3.906	<0.001	*Z* = −6.866	<0.001
Social activities	0.498 (0.379)	0.892 (0.511)	0.707 (0.449)	*Z* = −2.306	0.021	*Z* = −5.774	<0.001
Adventure activities	0.067 (0.078)	0.353 (0.251)	0.323 (0.203)	*Z* = −3.432	<0.001	*Z* = −7.326	<0.001

ADHD, attention deficit hyperactivity disorder; TDC, typically developing children; SD, standard deviation; MPH, methylphenidate; M-W test, Wilcoxon signed-rank test.

### 3.2 Behavioral performance in the go/no-go task

Behavioral performance on the go/no-go task was assessed using three primary metrics, which are summarized in Table 3. This table presents the accuracy rates for go and no-go trials and the reaction times for go trials for both the TDC and ADHD groups. At baseline, the ADHD group demonstrated significantly lower accuracy on both go and no-go trials relative to the TDC group. Post-MPH treatment, significant improvements were observed in the ADHD group, manifested as increased accuracy for both go and no-go trials and significantly reduced RT for go trial.

**TABLE 3:** Behavioral performance data related to response inhibition in the go/no-go task.

Behavioral data	TDC group	ADHD group					
		Post-MPH	Pre-MPH	TDC vs. Pre-MPH		Pre-MPH vs. Post-MPH	
	Mean (SD)	Mean (SD)	Mean (SD)	*t/*M-W test	*P*	*t/ Wilcoxon test*	*P*
Accuracy-go trial (%)	91.50 (8.17)	83.39 (12.53)	88.94 (8.16)	*Z* = −2.374	0.018	*Z* = −7.325	<0.001
Accuracy-no go trial (%)	90.67 (3.77)	85.44 (8.60)	90.47 (4.46)	*Z* = −1.791	0.073	*Z* = −7.377	<0.001
RT-go trial (ms)	454.167 (45.619)	474.722 (57.891)	456.389 (47.217)	*Z* = −1.481	0.139	*t* = 2.646	0.012

ADHD, attention deficit hyperactivity disorder; TDC, typically developing children; SD, standard deviation; MPH, methylphenidate; RT, reaction time.

### 3.3 Changes in Δ avg oxy-Hb concentration during the go/no-go task

**FIGURE 4:**
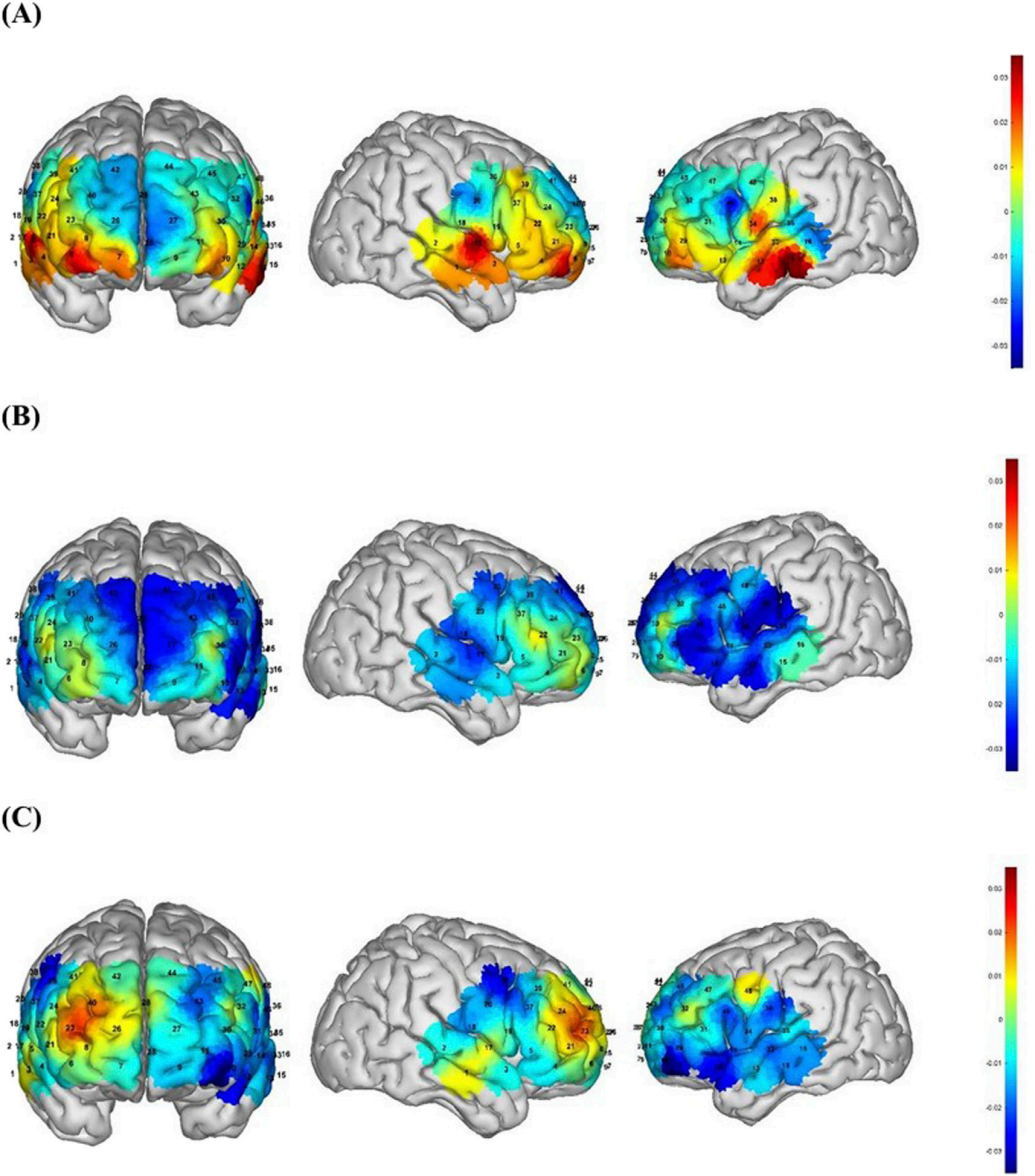
3D map of changes in △avg oxy-Hb concentration across 48 channels during the go/no-go task. **(A)** Front, left, and right views of △avg oxy-Hb concentration changes in the TDC group; **(B)** Front, left, and right views of △avg oxy-Hb concentration changes in the pre-MPH group; **(C)** Front, left, and right views of △avg oxy-Hb concentration changes in the post-MPH group. Color bar on the right: represents the level of activation, with red indicating higher activation and blue indicating lower activation.

**FIGURE 5:**
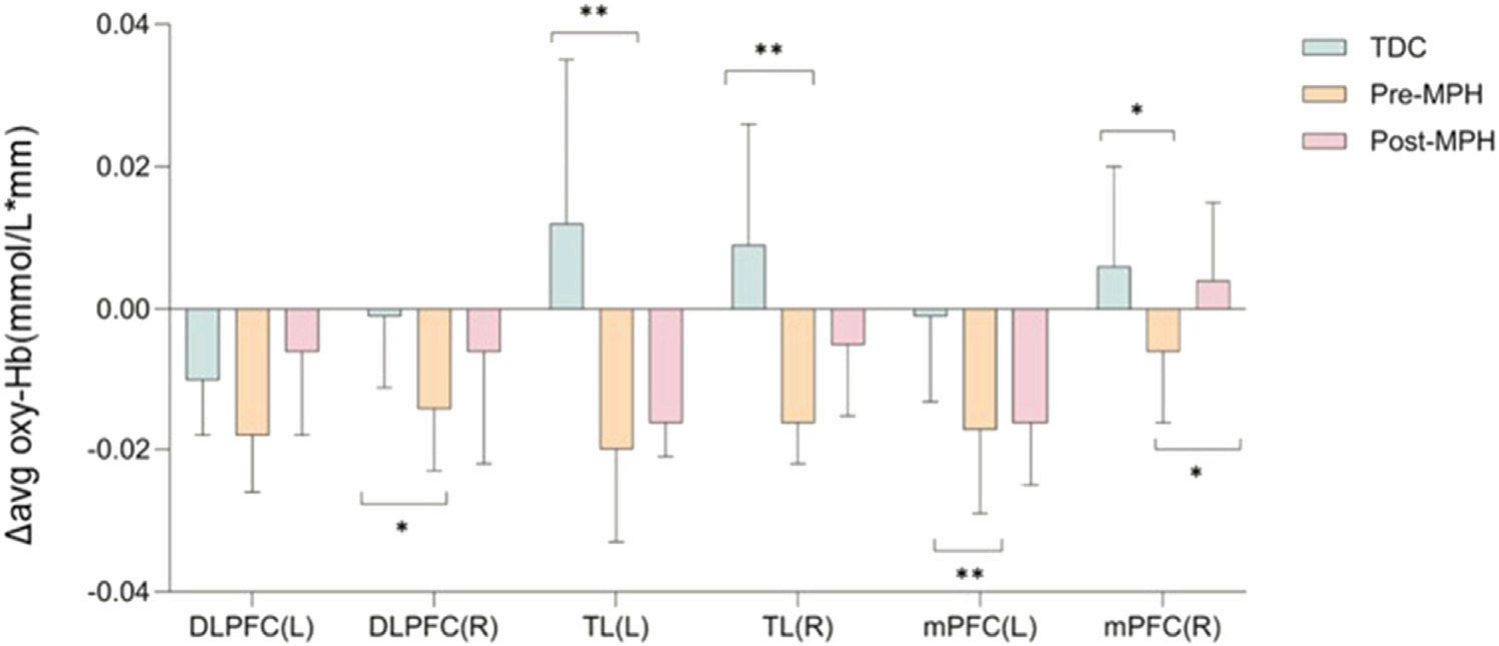
Hemodynamic changes during the performance of the go/no-go task. DLPFC, dorsolateral prefrontal cortex; mPFC, medial prefrontal cortex; TL, temporal lobe; R, right; L, left. *p < 0.05; **p < 0.01.

The Δ avg oxy-Hb concentrations for each channel during the go/no-go task are depicted for the TDC, pre-treatment ADHD, and post-treatment ADHD groups in Figures 4A–C, respectively. As shown by the ROI-based analysis (Figure 5), the TDC group demonstrated significantly greater Δ avg oxy-Hb concentrations relative to the pre-treatment ADHD group in the bilateral mPFC, right DLPFC, and bilateral TL. Moreover, post-MPH treatment was associated with a significant elevation of Δ avg oxy-Hb concentration in the right mPFC.

## 4 Discussion

This study aimed to provide an objective, multimodal evaluation of the therapeutic effects of MPH in children with ADHD. Recognizing the limitations of subjective clinical reports and the need for reliable biomarkers, we integrated clinical assessments, neuropsychological testing (the go/no-go task), and fNIRS to comprehensively assess treatment response. The study was motivated by the search for reliable neural biomarkers that could objectively track the response to MPH. Our central hypothesis was that, compared to TDC, children with ADHD would exhibit deficits at baseline in clinical symptoms, response inhibition, and prefrontal cortex activation. Furthermore, we posited that MPH treatment would not only alleviate core symptoms and ameliorate executive function deficits (i.e., response inhibition), but also enhance activation in key brain regions, namely the prefrontal cortex. Consistent with this hypothesis, our findings indicate the following conclusions.

### 4.1 Treatment with MPH can improve clinical symptoms and daily functional impairment in children with ADHD

The present study’s clinical findings align with the established efficacy of MPH as a first-line treatment for ADHD. At baseline, the ADHD group exhibited significantly higher symptom severity and functional impairment than the TDC group, consistent with the core diagnostic profile of the disorder. Following MPH intervention, we observed significant decreases in both SNAP-IV and WFIRS scores, approaching the levels of our baseline typically developing control TDC group. This result corroborates a large body of evidence demonstrating MPH’s effectiveness in ameliorating core symptoms and improving daily functioning (Cerrillo-Urbina et al., 2018; Ching et al., 2019; Cortese et al., 2018; Faraone et al., 2015; Fu et al., 2022; Groom and Cortese, 2022; Güven et al., 2019; Hawk et al., 2018; Kaalund-Brok et al., 2021; Li et al., 2021; Posner et al., 2020; Storebø et al., 2023; Yukifumi, 2018; Zheng et al., 2025). Although our findings strongly demonstrate the significant efficacy of MPH in treating ADHD, we cannot quantify the precise proportion of improvement attributable to development versus medication. MPH actions include dopamine and norepinephrine transporter inhibition, agonist activity at the serotonin type 1A receptor, and redistribution of the vesicular monoamine transporter 2 (Faraone, 2018). By enhancing the impact of dopamine and norepinephrine, psychostimulants increase the efficiency of prefrontal cortex activity and optimize executive and attentional function in patients suffering from ADHD (Arnsten, 2011; Arnsten and Pliszka, 2011; Ching et al., 2019; Cortese et al., 2017b; Pitzianti et al., 2020; Tucha et al., 2006; Vertessen et al., 2022; Wu et al., 2021).

While our results showed a robust clinical improvement, previous research has suggested several factors possibly influencing pharmacological treatment effects such as the predominant presentations of ADHD (Beery et al., 2017; Chazan et al., 2011; Vallejo-Valdivielso et al., 2019), comorbidities (Chan et al., 2017), intelligence quotient (Kaalund-Brok et al., 2021; Vallejo-Valdivielso et al., 2019) and ADHD symptom severity (Kaalund-Brok et al., 2021; Park et al., 2013) may moderate the real-world efficacy of MPH (Carucci et al., 2021). Taking academic-related outcomes as an example, a meta-analysis including 34 trials found that methylphenidate had only small and inconsistent effects on academic performance, improving general productivity, and accuracy in mathematics, but not reading (de Faria et al., 2022; Kortekaas-Rijlaarsdam et al., 2019; Martin, Collie, Roberts and Nassar, 2018).

A recent large prospective cohort study found that no biological, clinical, or cognitive factors could reliably predict pharmacological treatment outcomes in children and adolescents. To date, clear predictors of treatment response to ADHD medication remain elusive (Lilja et al., 2025). Given these circumstances, it is necessary to adopt a new perspective to determine which children are most likely to benefit from pharmacological treatment for ADHD. From a clinical standpoint, we raise ethical concerns about withholding medication from children and adolescents who are not yet diagnosed with ADHD or do not meet diagnostic criteria but exhibit severe functional impairments. Conversely, an ADHD diagnosis alone is not sufficient to automatically warrant medication. Future research should explore individualized precision pharmacotherapy.

### 4.2 Behavioral performance for go/no-go task

The behavioral findings from the go/no-go task demonstrated that, relative to TDC, children with ADHD showed significantly compromised accuracy on both go and no-go trials. This pattern of results is broadly consistent with a large body of previous research documenting poorer overall performance on the go/no-go task among children with ADHD compared to their healthy counterparts (Cheng et al., 2025; Li et al., 2025; Metin et al., 2012; Monden et al., 2012a; Münger et al., 2021), with accuracy being the most prominent indicator of this impairment (Monden et al., 2012b). However, some studies have found no significant difference in behavioral performance between children with ADHD and the TDC (Miao et al., 2017; Nagashima et al., 2014b; Smith et al., 2006; Wu et al., 2023; Zhu et al., 2023). It has been postulated that during early development, typically developing children exhibit a more robust maturation of control systems, while children with ADHD follow an atypical developmental trajectory, particularly in the domain of impulse regulation (Barkley, 1997). This divergence in developmental pathways may underlie the observed behavioral discrepancies (Miao et al., 2017). Following MPH treatment, children with ADHD showed improved accuracy on both go and no-go trials and a significant reduction in go RT, indicating significant improvements in their response inhibition and attentional deficits. These findings are consistent with a large body of previous research, showing that MPH treatment improves the performance of children with ADHD on go/no-go task (Coghill et al., 2007; Li et al., 2021; Monden et al., 2012a; Monden et al., 2012b).

### 4.3 Right mPFC activation as an objective neuro-functional biomarker for assessing the efficacy of methylphenidate

In the present study, we observed that, at baseline, children with ADHD exhibited significantly diminished activation in the bilateral mPFC, right DLPFC, and bilateral TL compared to the TDC group. This finding of prefrontal hypoactivation corroborates a substantial body of prior evidence indicating that children with ADHD consistently show under activation in frontal areas during response inhibition tasks, encompassing the prefrontal cortex, right inferior frontal gyrus, and right middle frontal gyrus (Inoue et al., 2012; Kim et al., 2023; Y. Li et al., 2024; Miao et al., 2017; Monden et al., 2012a; Monden et al., 2015; T. Wu et al., 2023; Xiao et al., 2012; Yu et al., 2022; Zhu et al., 2023). The convergence of evidence from qualitative fNIRS analyses and fMRI meta-analyses further supports these patterns of hypoactivation (Cubillo et al., 2012; Hart et al., 2013). Notably, prior fMRI investigations have corroborated these findings by demonstrating significantly reduced cerebral blood flow within the frontal lobes of children with ADHD during the execution of inhibitory tasks (Passarotti et al., 2010; Rubia et al., 2010). Crucially, our findings revealed that a course of MPH treatment resulted in a significant augmentation of activation within the right mPFC in the ADHD cohort. This observation is in agreement with previous studies that have also documented a significant enhancement of prefrontal cortical activation following MPH administration (Chou et al., 2015; Ishii-Takahashi et al., 2015; Kawai et al., 2021; Li et al., 2021; Monden et al., 2012a; Monden et al., 2012b; Nagashima et al., 2014a; Rubia et al., 2014; Rubia et al., 2011). However, it is important to note that the literature is not entirely consistent. For instance, Nakanishi et al. found that MPH treatment did not increase prefrontal cortex activation. Such discrepancies across studies may be attributed to variations in methodological factors, including the cognitive tasks employed, drug dosages, patient ages, and treatment durations. Therefore, our finding that MPH induces increased activation in the right mPFC provides new and compelling evidence supporting the efficacy of MPH in modulating prefrontal function in ADHD (Nakanishi et al., 2017).

Functioning as the supreme integrative hub of the central nervous system, the PFC is pivotal in governing cognitive regulation, behavioral control, and socio-emotional processing. Its engagement spans executive functions (such as working memory and goal-directed behavior), the integration of emotion and cognition (including risk assessment and moral decision-making), and the modulation of social behaviors (encompassing impulse inhibition and reward processing). This enhancement consequently contributes to the amelioration of ADHD’s core symptomatology across multiple domains: it improves attention, diminishes hyperactivity, mitigates deficits in daily functioning (e.g., learning and social skills), and augments impulse control (Cortese et al., 2017b). Ultimately, this leads to a further elevation of cognitive performance in children with ADHD (Coghill et al., 2014; C. S. Wu et al., 2021).

### 4.4 Clinical utility

First, the primary benefit of an objective assessment tool like fNIRS is to complement and, in some cases, surpass the limitations of subjective rating scales. Parent and teacher reports, while essential, can be influenced by rater bias, expectations, and differing interpretations of behavior. fNIRS provides a direct, quantifiable measure of cortical hemodynamic activity, offering an unbiased “biological readout” of brain function. This objectivity is crucial for establishing a more reliable baseline and for measuring treatment-induced changes with greater precision. Second, fNIRS offers a significant advantage in quantifying clinical improvements, particularly for subtle or “hard-to-perceive” functions. For instance, while parents may observe an amelioration of inattention, hyperactivity, or impulsivity following MPH treatment, fNIRS can precisely quantify changes in cortical activation. This enables us to move beyond a simple dichotomy of “improved versus not improved” and measure the degree of neurophysiological change, providing a more nuanced, sensitive, and objective metric of treatment efficacy, thereby enhancing medication adherence. Third, our findings lay the groundwork for developing predictive models for children with ADHD. The baseline hemodynamic patterns we identified could serve as potential biomarkers. For instance, children with a specific pattern of prefrontal hypoactivation may exhibit a more robust response to MPH. Future studies with larger cohorts are needed to utilize leverage baseline fNIRS data to develop algorithms that can identify children likely to have a neurophysiological response to MPH, while also predicting the magnitude of clinical improvement based on initial brain activation patterns. This represents a highly promising and clinically relevant direction for future research.

A key challenge for the future lies in bridging the gap between the neurobiological-level effects observed with fNIRS and the underlying molecular mechanisms of MPH. While our study provides a non-invasive window into the functional outcomes of MPH administration, it does not directly measure its primary molecular actions, such as the blockade of DA and NE transporters. We propose that the hemodynamic signatures identified in our study could serve as crucial intermediate phenotypes, or “neurophenotypes,” linking molecular genetics to clinical outcomes. For instance, future multimodal research integrating fNIRS with genotyping could investigate whether specific genetic polymorphisms in dopaminergic or noradrenergic pathways predict the magnitude of prefrontal activation observed post-MPH administration. Such an approach would be instrumental in advancing the field of ADHD toward a truly personalized medicine paradigm, wherein treatment decisions are informed by an integrated understanding of a patient’s molecular profile, brain function, and behavioral symptoms.

## 5 Limitations

Several limitations of the present study should be acknowledged. First, the sample size of this study was relatively small because we excluded children with other common neurodevelopmental disorders to minimize their impact on the results. Additionally, a potential limitation of this study is the imbalance in group sizes, which may have affected the statistical power. However, the statistical methods used are robust to this issue, and the significant effects observed suggest a meaningful impact of the intervention. Therefore, future research should include a larger sample size and optimize the sample structure (e.g., by including subjects with different clinical subtypes, genders, medication dosages, and comorbidities) to enhance the reliability and external validity of the results. It should be noted that an adult MNI template was used for anatomical localization in our pediatric sample. While this is a widely adopted approach in the fNIRS field, future studies would benefit from using age-appropriate brain templates or neuronavigation with individual MRI scans to further improve localization accuracy. Furthermore, this study did not include a placebo control group and only had a single follow-up time point at 6 months post-treatment, which meant we were unable to rule out the influence of outcome variability over different follow-up periods. Future research is recommended to adopt a multi-center, double-blind, randomized controlled trial (RCT) design with additional follow-up time points at 1.5 h, 3 months, 6 months, and 1 year post-administration to further investigate the neuroimaging mechanisms and objective biomarkers of MPH’s therapeutic effects in children with ADHD.

## 6 Conclusion

In this study, we obtained the following findings: First, the activation of the right mPFC can serve as an objective neurofunctional biomarker for assessing the efficacy of MPH treatment using fNIRS. Concurrently, the multimodal approach, which combines clinical assessments, neuropsychological testing (go/no-go task), and fNIRS, represents a highly promising technique for the objective evaluation of MPH’s therapeutic effects in children with ADHD.

## Data Availability

The original contributions presented in the study are included in the article/Supplementary Material, further inquiries can be directed to the corresponding author.
