# Supplementary material for: Effects of methylphenidate on children with attention deficit hyperactivity disorder: a study using clinical and multimodal approaches including go/no-go task and functional near-infrared spectroscopy
Source: Front Pharmacol. 2025 Dec 8;16:1726716. doi: 10.3389/fphar.2025.1726716 (PMC12723010; doi:10.3389/fphar.2025.1726716)
Supplement: Supplementary file 1 [file DataSheet1.pdf]

Supplementary Material

Brodmann areas and brain regions related to fNIRS channels

fNIRS Channels	Brodmann Area (BA), Brain Region	Percentage of Overlap
CH01	21 - Middle Temporal gyrus	0.8053
	22 - Superior Temporal Gyrus	0.1947
CH02	2 - Primary Somatosensory Cortex	0.0124
	21 - Middle Temporal gyrus	0.1025
	22 - Superior Temporal Gyrus	0.8789
	48 - Retrosubicular area	0.0062
CH03	6 - Pre-Motor and Supplementary Motor Cortex	0.0475
	38 - Temporopolar area	0.4237
	44 - pars opercularis_ part of Broca's area	0.0576
	45 - pars triangularis Broca's area	0.0712
	48 - Retrosubicular area	0.4
CH04	45 - pars triangularis Broca's area	0.4573
	46 - Dorsolateral prefrontal cortex	0.5427
CH05	44 - pars opercularis_ part of Broca's area	0.0654
	45 - pars triangularis Broca's area	0.9346
CH06	10 - Frontopolar area	0.8296
	11 - Orbitofrontal area	0.0926
	46 - Dorsolateral prefrontal cortex	0.0778
CH07	10 - Frontopolar area	1
CH08	9 - Dorsolateral prefrontal cortex	0.0193
	10 - Frontopolar area	0.7606
	46 - Dorsolateral prefrontal cortex	0.2201
CH09	10 - Frontopolar area	0.9671
	11 - Orbitofrontal area	0.0329
CH10	10 - Frontopolar area	0.6762
	46 - Dorsolateral prefrontal cortex	0.3238
CH11	10 - Frontopolar area	0.5119
	46 - Dorsolateral prefrontal cortex	0.4881
CH12	45 - pars triangularis Broca's area	0.3741
	46 - Dorsolateral prefrontal cortex	0.6259
CH13	21 - Middle Temporal gyrus	0.4689
	38 - Temporopolar area	0.4469
	48 - Retrosubicular area	0.0842
CH14	6 - Pre-Motor and Supplementary Motor Cortex	0.0278
	44 - pars opercularis_ part of Broca's area	0.3125
	45 - pars triangularis Broca's area	0.559
	48 - Retrosubicular area	0.1007
CH15	20 - Inferior Temporal gyrus	0.0127

	21 - Middle Temporal gyrus	0.9299
	22 - Superior Temporal Gyrus	0.0573
CH16	21 - Middle Temporal gyrus	0.1786
	22 - Superior Temporal Gyrus	0.8214
CH17	6 - Pre-Motor and Supplementary Motor Cortex	0.2825
	22 - Superior Temporal Gyrus	0.0889
	43 - Subcentral area	0.4063
	48 - Retrosubicular area	0.2222
CH18	1 - Primary Somatosensory Cortex	0.1366
	2 - Primary Somatosensory Cortex	0.3571
	43 - Subcentral area	0.5062
CH19	4 - Primary Motor Cortex	0.0393
	6 - Pre-Motor and Supplementary Motor Cortex	0.6286
	43 - Subcentral area	0.0393
	44 - pars opercularis_ part of Broca's area	0.2929
CH20	1 - Primary Somatosensory Cortex	0.1901
	3 - Primary Somatosensory Cortex	0.2183
	4 - Primary Motor Cortex	0.2958
	6 - Pre-Motor and Supplementary Motor Cortex	0.1549
	43 - Subcentral area	0.1408
CH21	45 - pars triangularis Broca's area	0.3068
	46 - Dorsolateral prefrontal cortex	0.6932
CH22	44 - pars opercularis_ part of Broca's area	0.0902
	45 - pars triangularis Broca's area	0.8078
	46 - Dorsolateral prefrontal cortex	0.102
CH23	9 - Dorsolateral prefrontal cortex	0.3774
	46 - Dorsolateral prefrontal cortex	0.6226
CH24	9 - Dorsolateral prefrontal cortex	0.8423
	44 - pars opercularis_ part of Broca's area	0.0315
	45 - pars triangularis Broca's area	0.0541
	46 - Dorsolateral prefrontal cortex	0.0721
CH25	10 - Frontopolar area	1
CH26	9 - Dorsolateral prefrontal cortex	0.8805
	10 - Frontopolar area	0.1195
CH27	9 - Dorsolateral prefrontal cortex	0.877
	10 - Frontopolar area	0.0984
	46 - Dorsolateral prefrontal cortex	0.0246
CH28	8 - Includes Frontal eye fields	0.3068
	9 - Dorsolateral prefrontal cortex	0.6932
CH29	45 - pars triangularis Broca's area	0.4661
	46 - Dorsolateral prefrontal cortex	0.5339
CH30	9 - Dorsolateral prefrontal cortex	0.0987
	45 - pars triangularis Broca's area	0.1211

	46 - Dorsolateral prefrontal cortex	0.7803
CH31	44 - pars opercularis_ part of Broca's area	0.1326
	45 - pars triangularis Broca's area	0.8674
CH32	9 - Dorsolateral prefrontal cortex	0.4414
	44 - pars opercularis_ part of Broca's area	0.2252
	45 - pars triangularis Broca's area	0.2252
	46 - Dorsolateral prefrontal cortex	0.1081
CH33	6 - Pre-Motor and Supplementary Motor Cortex	0.0234
	22 - Superior Temporal Gyrus	0.4415
	43 - Subcentral area	0.204
	48 - Retrosubicular area	0.3311
CH34	4 - Primary Motor Cortex	0.0213
	6 - Pre-Motor and Supplementary Motor Cortex	0.5674
	43 - Subcentral area	0.344
	44 - pars opercularis_ part of Broca's area	0.0674
CH35	1 - Primary Somatosensory Cortex	0.0162
	2 - Primary Somatosensory Cortex	0.5566
	22 - Superior Temporal Gyrus	0.1036
	42 - Primary and Auditory Association Cortex	0.0032
	43 - Subcentral area	0.2071
	48 - Retrosubicular area	0.1133
CH36	1 - Primary Somatosensory Cortex	0.3544
	2 - Primary Somatosensory Cortex	0.1754
	3 - Primary Somatosensory Cortex	0.193
	4 - Primary Motor Cortex	0.0246
	43 - Subcentral area	0.2526
CH37	6 - Pre-Motor and Supplementary Motor Cortex	0.1423
	9 - Dorsolateral prefrontal cortex	0.3933
	44 - pars opercularis_ part of Broca's area	0.4644
CH38	4 - Primary Motor Cortex	0.351
	6 - Pre-Motor and Supplementary Motor Cortex	0.649
CH39	6 - Pre-Motor and Supplementary Motor Cortex	0.127
	8 - Includes Frontal eye fields	0.131
	9 - Dorsolateral prefrontal cortex	0.7421
CH40	8 - Includes Frontal eye fields	0.1167
	9 - Dorsolateral prefrontal cortex	0.8833
CH41	8 - Includes Frontal eye fields	0.954
	9 - Dorsolateral prefrontal cortex	0.046
CH42	8 - Includes Frontal eye fields	1
CH43	8 - Includes Frontal eye fields	0.0356
	9 - Dorsolateral prefrontal cortex	0.9644
CH44	8 - Includes Frontal eye fields	0.9851
	9 - Dorsolateral prefrontal cortex	0.0149
CH45	8 - Includes Frontal eye fields	0.5649

	9 - Dorsolateral prefrontal cortex	0.4351
CH46	6 - Pre-Motor and Supplementary Motor Cortex	0.4103
	9 - Dorsolateral prefrontal cortex	0.0696
	44 - pars opercularis_ part of Broca's area	0.5201
CH47	6 - Pre-Motor and Supplementary Motor Cortex	0.3544
	9 - Dorsolateral prefrontal cortex	0.6456
CH48	3 - Primary Somatosensory Cortex	0.2078
	4 - Primary Motor Cortex	0.3922
	6 - Pre-Motor and Supplementary Motor Cortex	0.4
